# Seasonal SUHI Analysis Using Local Climate Zone Classification: A Case Study of Wuhan, China

**DOI:** 10.3390/ijerph18147242

**Published:** 2021-07-06

**Authors:** Lingfei Shi, Feng Ling, Giles M. Foody, Zhen Yang, Xixi Liu, Yun Du

**Affiliations:** 1 College of Resources and Environmental Sciences, Henan Agricultural University, Zhengzhou 450002, China; shilingfei14@mails.ucas.ac.cn; 2 Innovation Academy for Precision Measurement Science and Technology, Chinese Academy of Sciences, Wuhan 430077, China; duyun@whigg.ac.cn; 3 School of Geography, University of Nottingham, Nottingham NG7 2RD, UK; giles.foody@nottingham.ac.uk; 4 College of Information Science and Engineering, Henan University of Technology, Zhengzhou 450001, China; zhenyang@haut.edu.cn (Z.Y.); liuxixi@haut.edu.cn (X.L.)

**Keywords:** surface urban heat island, local climate zone, land surface temperature

## Abstract

The surface urban heat island (SUHI) effect poses a significant threat to the urban environment and public health. This paper utilized the Local Climate Zone (LCZ) classification and land surface temperature (LST) data to analyze the seasonal dynamics of SUHI in Wuhan based on the Google Earth Engine platform. In addition, the SUHI intensity derived from the traditional urban–rural dichotomy was also calculated for comparison. Seasonal SUHI analysis showed that (1) both LCZ classification and the urban–rural dichotomy confirmed that Wuhan’s SHUI effect was the strongest in summer, followed by spring, autumn and winter; (2) the maximum SUHI intensity derived from LCZ classification reached 6.53 °C, which indicated that the SUHI effect was very significant in Wuhan; (3) LCZ 8 (i.e., large low-rise) had the maximum LST value and LCZ G (i.e., water) had the minimum LST value in all seasons; (4) the LST values of compact high-rise/midrise/low-rise (i.e., LCZ 1–3) were higher than those of open high-rise/midrise/low-rise (i.e., LCZ 4–6) in all seasons, which indicated that building density had a positive correlation with LST; (5) the LST values of dense trees (i.e., LCZ A) were less than those of scattered trees (i.e., LCZ B) in all seasons, which indicated that vegetation density had a negative correlation with LST. This paper provides some useful information for urban planning and contributes to the healthy and sustainable development of Wuhan.

## 1. Introduction

It was reported by the United Nations that 68% of the world’s population will be living in cities by 2050 [1]. The huge population in the city will bring a more rapid and intensive urbanization process, which will significantly change the city, regional and even global ecological environment [2]. Among urban environmental problems, the urban heat island (UHI) is the most obvious one for all large urban centers [3,4,5]. It has caused a number of urban problems involving heatwaves and air pollution [6,7,8,9], energy utilization [10], air quality [11,12], urban hydrology [12], physical and chemical properties of urban soil [13], biological spatial distribution and behavioral activities [14] and human health and comfort [10,15]. 

UHI is the phenomenon in which an urban area is warmer than its surrounding rural area [16,17]. It has been reported for cities and regions worldwide [18], often with local field sites that are extremely diverse in their physical and climatological characteristics. These sites are usually described only as “urban area” or “rural area”, leaving much uncertainty about the actual exposure and land cover of the sites [19,20]. This uncertainty surrounding urban and rural areas brings difficulties in assessing the SUHI intensity [21]. To address this problem, the Local Climate Zone (LCZ) classification scheme was designed by Stewart and Oke in 2012 [22]. The LCZ classification scheme intends to be universal and applicable in cities all over the world, offering the possibility to compare different areas of different cities with trenchant distinctions representing the heterogeneous thermal behavior in an urban environment. It has been widely used for urban climate studies [23,24,25,26,27].

To quantify the UHI effect, the temperature difference in the urban and rural area is defined as the UHI intensity. In general, both air temperature and land surface temperature (LST) can be used to calculate UHI intensity. Air temperature is measured by interpolation with standard meteorological stations or ground-based air temperature measurements. LST is produced by the thermal infrared bands from remote sensing images, which can effectively depict the spatially continuous thermal environment patterns better than meteorological stations [28,29]. Moreover, remote sensing images have the characteristics of wide coverage and a short acquisition period, which contribute to the production of rapid and accurate LST maps [30,31]. Hence, remote sensing technology has become a powerful tool in UHI analysis. Single-channel, multi-channel, multi-angle, multi-temporal and hyperspectral retrieval methods have been employed in LST retrieval based on remote sensing imagery.

To distinguish UHI intensity using the air temperature difference, the UHI intensity based on the LST difference is defined as the surface urban heat island (SUHI) intensity [4,32]. 

Based on the LCZ classification scheme, the SUHI intensity is regarded as the LST difference of any two LCZ classes [22]. In practical application, Alexander et al. (2014) employed the LST of compact midrise (i.e., LCZ 2) and low plants (i.e., LCZ D) to calculate the SUHI intensity [33]. Leconte et al. (2015) also used the LST of LCZ 2 and LCZ D to obtain the SUHI intensity [34]. Furthermore, Lin et al. (2016) selected the LST of compact LCZ 2 and LCZ D to calculate the SUHI intensity of Fuzhou city [20]. Ng (2015) regarded the LST difference of open high-rise (i.e., LCZ 4) and sparsely built (i.e., LCZ 9) as the SUHI intensity of Singapore [35]. Bechtel et al. (2019) used LCZ to perform SUHI analysis for 50 cities across the globe [36]. However, most of these studies only sought to calculate the SUHI intensity with the LCZ classification scheme at a certain time, neglecting any analysis of the seasonal SUHI effect.

Seasonal climate change results in different SUHI effects. Some researchers have studied seasonal SUHI using two methods. The first is in terms of the traditional urban–rural dichotomy method. Wu et al. (2019) examined the seasonal effect of SUHI in Dalian, a coastal city in China [37]; Yuan et al. (2017) analyzed the seasonal variations in SUHI in Shenzhen [38]; Schatz et al. (2014) characterized the seasonality of the SUHI effect in Madison, Wisconsin [39]; Haashemi et al. (2016) investigated the seasonal variability of SUHI in the Tehran metropolitan area, Iran [40]. The second is in terms of the LCZ classification method. Budhiraja et al. (2019) evaluated the seasonal SHUI intensities in Delhi using LCZ classification [41]; Du et al. (2020) used an LCZ map to analyze the seasonal variation in LST in the Nanjing urban area [42]. Geletic et al. (2019) analyzed the inter- and intra-zonal seasonal variability of SUHI based on LCZ classification in three central European cities (i.e., Prague, Brno, and Novi Sad) [43]. Moreover, the LST maps used by Budhiraja et al. (2019) and Du et al. (2020) had coarse spatial resolution and single temporality, respectively. This lowers the accuracy of SHUI intensity calculation. Generally, studies about seasonal SUHI using LCZ classification are scarce so far.

Affected by the SUHI effect, there has been an increasing number of “furnace cities” in China [44]. The terms “furnace cities” refers literally to cities resembling a furnace, which indicates that these cities are extremely hot in summer. As one of the well-established furnace cities in China, Wuhan’s SUHI effect is always significant. Some researchers have studied the SUHI effect in Wuhan. Shen et al. (2016) employed a long-term (26-years) summer LST data series to analyze the SUHI effect in Wuhan [28]. Zhang et al. (2012) used the normalized difference vegetation index to study the SUHI effect in Wuhan [45]. Ren et al. (2007) monitored the temporal changes in SUHI intensity in Wuhan [46]. Wu et al. (2014) assessed the effects of land use on the SUHI effect in Wuhan [47]. Wuhan has experienced rapid urbanization in recent decades [48], which has further intensified the urban heat island effect. With the increasing research interest in LCZ classification, some researchers have studied the LCZ classification of Wuhan [49,50]. However, the subsequent and most important SUHI analysis using the LCZ classification of Wuhan has not yet been conducted. Hence, the purpose of this paper is two-fold: (1) to analyze the urban thermal environment using LCZ classification of Wuhan; (2) to analyze and compare the seasonal SUHI intensities derived from two methods: traditional urban–rural dichotomy and LCZ classification. 

Section 1 has introduced this paper, and the remainder is organized as follows: Section 2 introduces the study area and datasets. Section 3 describes the methods used for SUHI analysis. Section 4 analyzes the seasonal SUHI effect, and Section 5 summarizes the conclusions and areas for further improvement.

## 2. Study Area and Datasets

### 2.1. Study Area

**Figure 1:**
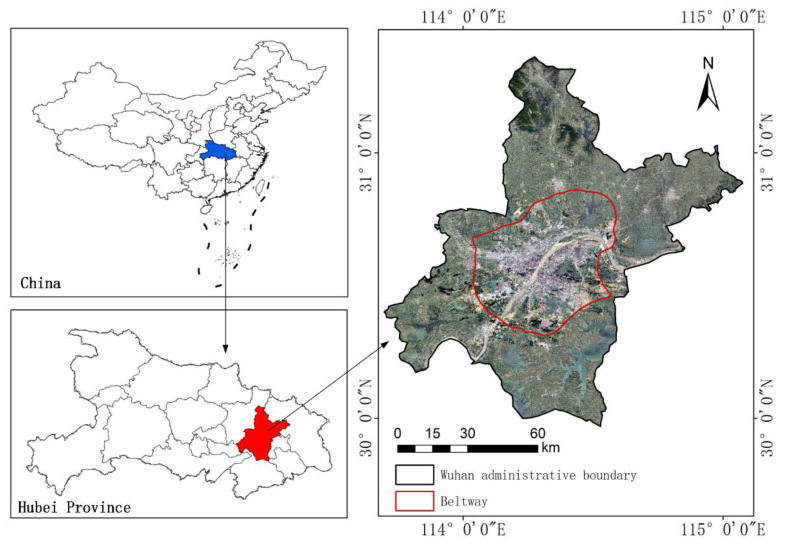
The location of Wuhan.

The city of Wuhan, China, was selected as the study area, as shown in Figure 1. It is situated in Central China, at latitude 29°58′–31°22′ N and longitude 113°41′–115°05′ E. Wuhan covers 8569.15 km^2^ and the existing water surface area accounts for 24.9% of the entire city’s area [51]. It has a hot and rainy summer, which is affected by the tropical ocean monsoon, and a cold and wet winter, affected by the Siberian winter monsoon. Wuhan is also one of the largest cities in China, with a population of more than 10 million. The UHI effect has strong effects on human health, so it is an important area of study in Wuhan. 

### 2.2. LCZ Classification of Wuhan

As shown in Table 1, the LCZ classification scheme proposed by Stewart and Oke (2012) consisted of 17 LCZ classes. Of these, 13 are encountered in Wuhan: 7 (LCZ 1, LCZ 2, LCZ 3, LCZ 4, LCZ 5, LCZ 6, and LCZ 8) are of the built type and 6 (LCZ A, LCZ B, LCZ D, LCZ E, LCZ F, and LCZ G) belong to the land cover type. Each LCZ class represents a simple combination of buildings, roads, plants, soils, rock, and water with different mounts.

**Figure 2:**
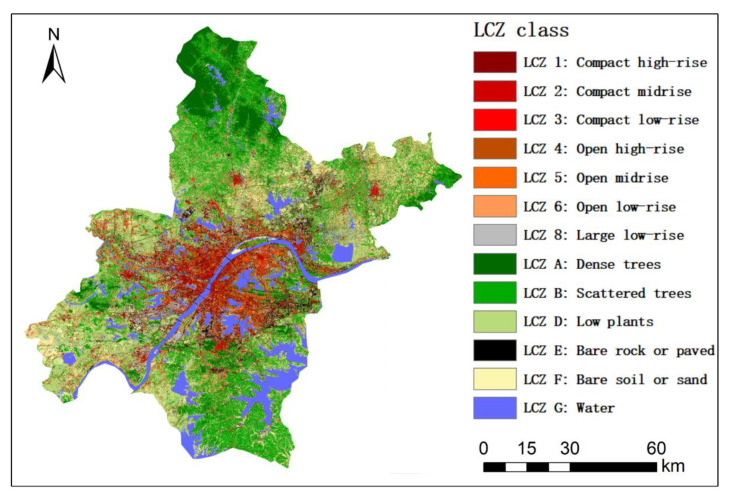
Local Climate Zone (LCZ) classification of Wuhan in 2018 [49]. Used with permission.

Shi et al. (2021) used multi-source free available datasets and the Google Earth Engine (GEE) platform to generate a series of LCZ classifications of Wuhan city in 2018 [49]. The LCZ classification with the highest overall accuracy was used here (Figure 2). The datasets used to generate this LCZ classification were seasonal Sentinel-2 and Sentinel-1 data, the texture information and spectral indices derived from Sentinel-2 data, and OpenStreetMap (OSM) data. Except for the OSM dataset, other datasets were raster data with 10 m spatial resolution. Hence, the OSM dataset was converted to raster data with 10 m spatial resolution to ensure consistency with the other datasets. Then, these datasets were combined and classified using the random forest method to generate a 10 m LCZ classification map of Wuhan.

The confusion matrix of this LCZ classification is shown in Table 2. The overall accuracy (OA), producer’s accuracy (PA), and user’s accuracy (UA) were used to assess the accuracy of the LCZ map in Wuhan. OA refers to the proportion of correctly classified samples in the total number of samples—in other words, the sum of all the values of the diagonal in the confusion matrix divided by the sum of all the samples. PA and UA are the accuracies for a certain LCZ class. In particular, PA and UA can be calculated as the number of correctly classified LCZ class samples divided by the total number of this LCZ class in the ground truth or map, respectively. The OA value is 76.64%, which meets the application requirements in this study. OAb and OAlc represent OA for built-type LCZ classes and land-cover-type LCZ classes, respectively. The OAb value is lower than the OAlc value, which indicates that built type LCZ classes were more difficult to distinguish than land-cover-type LCZ classes. 

### 2.3. The Remote Sensing Data Used for LST Retrieval

Landsat 8 images were selected to generate LST maps of Wuhan based on the GEE platform. Landsat 8 launched on 11 February 2013. It carries two science instruments: the Operational Land Imager (OLI) and the Thermal Infrared Sensor (TIRS). The OLI instrument provides 8 spectral bands (i.e., visible, NIR, SWIR) at a spatial resolution of 30 m and 1 panchromatic band at a spatial resolution of 15 m. The TIRS instrument provides 2 thermal bands at a spatial resolution of 100 m. 

GEE is a powerful cloud computing platform for petabytes of publicly available remote sensing imagery processing and analysis. The LST maps were generated using the code provided by Ermida et al. (2020) (https://code.earthengine.google.com/?accept_repo=users/sofiaermida/landsat_smw_lst accessed on 3 July 2021) [52]. The statistical mono-window algorithm was employed in this code because it was easy to calibrate and implement. To ensure consistency with the spatial resolution of LCZ classification, the LST images were resampled to 10 m using the bilinear interpolation method in the GEE platform. 

The quantity of available Landsat images in 2018 was limited for LST retrieval in each season. Cloud contamination is a major factor affecting the use of Landsat images, further lowering the quantity of Landsat images used for LST retrieval. To obtain representative LST maps for each season, this study widened the time range of LST retrieval (i.e., 2017 to 2019). In addition, the cloud coverage condition was set as less than 30%. After selecting all qualifying Landsat images for each season, all of them were first converted to LST images. Then, the median value of these LST images was calculated to produce the seasonal LST map. 

## 3. Methods

**Figure 3:**
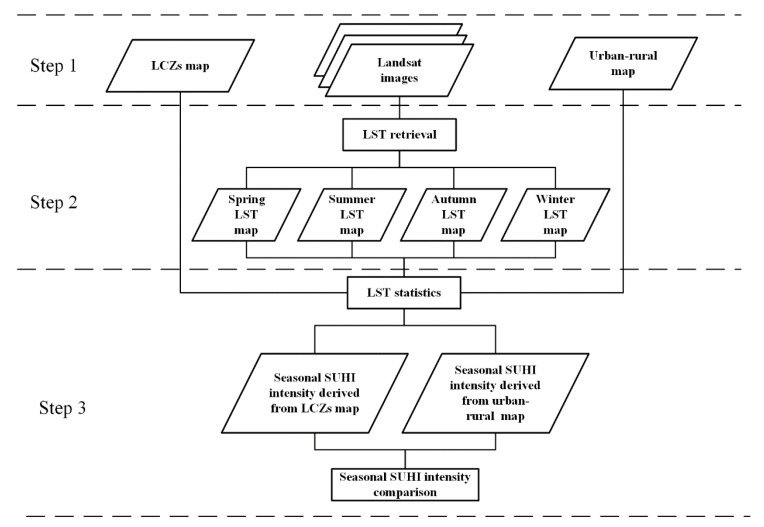
The flowchart of seasonal SUHI analysis.

In this study, the seasonal SUHI analysis in Wuhan had three steps (Figure 3). The first step was data preparation. The second step was LST retrieval using GEE code to generate seasonal LST maps, and the last step was seasonal SUHI analysis using traditional urban–rural dichotomy and LCZ classification methods.

### 3.1. SHUI Analysis Using the LCZ Classification 

SUHI intensity using LCZ classification can be represented as the differences in the LST values for the different LCZ classes [22]. Based on LST maps and LCZ classification of Wuhan, the average LST of each LCZ class was calculated for SHUI intensity calculation. To gain a simple and intuitive understanding of SUHI intensity, this study focused on the average LST value difference per LCZ class compared to LCZ D (i.e., low plants). Then, the LST differences in other LCZ classes and the LCZ D class were calculated to represent SUHI intensities. The equation of SUHI intensity using LCZ classification was as follows:

(1) $${\mathrm{SUHI}}_{LCZ}={\mathrm{LST}}_{LCZ\;X}-{\mathrm{LST}}_{LCZ\;D}$$

where ${\mathrm{SUHI}}_{LCZ}$ is the SUHI intensity using LCZ classification; LCZ X represents any LCZ class other than LCZ D; ${\mathrm{LST}}_{LCZ\;X}$ and ${\mathrm{LST}}_{LCZ\;D}$ represent the average LST value of LCZ X and LCZ D, respectively.

### 3.2. SHUI Analysis Using the Urban–Rural Dichotomy 

SUHI intensity using the urban–rural dichotomy can be represented as the difference in the average temperature of the urban and rural area. In this study, the Wuhan beltway (Figure 1) was regarded as the boundary line. The area within the Wuhan beltway was regarded as the urban area, the area outside the Wuhan beltway was considered the rural area. As there are rivers or lakes in the urban center, the water in the urban area would be masked when calculating the average LST. The equation of SUHI intensity using the urban–rural dichotomy was as follows:

(2) $${\mathrm{SUHI}}_{urban-rural}={\mathrm{LST}}_{urban}-{\mathrm{LST}}_{rural}$$

where ${\mathrm{SUHI}}_{urban-rural}$ is the SUHI intensity using the urban–rural dichotomy; ${\mathrm{LST}}_{urban}$ and ${\mathrm{LST}}_{rural}$ are the average LST value of the urban and rural area, respectively. 

## 4. Results and Discussion

### 4.1. Seasonal LST Maps of Wuhan

**Figure 4:**
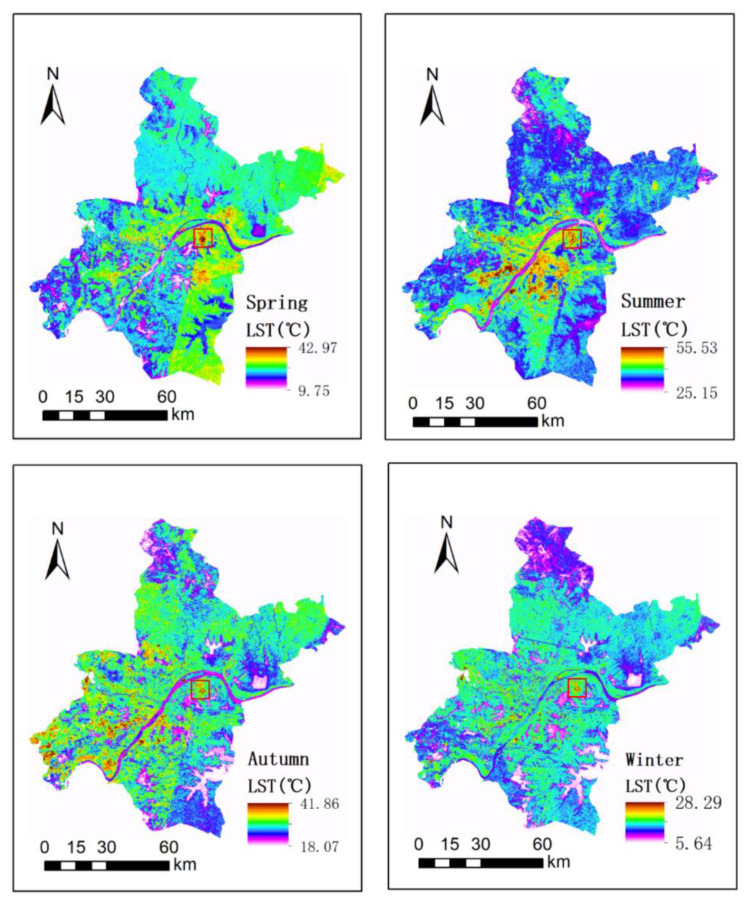
Seasonal LST maps of Wuhan.

Figure 4 shows the land surface temperature map of Wuhan in the different seasons. From Figure 4, it is clear that the summer LST image had the most significant LST difference between the urban center and its surrounding area, followed by spring, autumn, and winter LST images. Through comparing LCZ classification (Figure 2) and LST maps (Figure 4), it was found that most built-type LCZ classes (e.g., LCZ 1–6) were located in the urban center, where the LST values were relatively high. The area in the red box was large low-rise (i.e., LCZ 8), where many factories were clustered, according to investigation using Google Earth images. Large low-rise (i.e., LCZ 8) had a significantly high LST value in different seasons. A large amount of heat energy was released during industrial production, which exacerbated the SUHI effect. The land cover classes were distributed in the surrounding area of the urban center and had a relatively low LST. Water (i.e., LCZ G) had the lowest LST in all seasons, followed by dense trees (i.e., LCZ A), which indicated that water area and vegetation density could alleviate the SUHI effect effectively.

### 4.2. Seasonal SUHI Intensity Derived from LCZ Classification

Based on the LST images, all pixels in LCZ classification had corresponding LST values and the average LST of each LCZ class was calculated as shown in Table 3. As expected, the temperature was the highest in summer and lowest in winter, but there were clear associations with LCZ classes. The LST values of compact high-rise/midrise/low-rise (i.e., LCZ 1–3) were higher than those of open high-rise/midrise/low-rise (i.e., LCZ 4–6), which indicated that there was a positive correlation between building density and LST. The dense building areas (i.e., LCZ 1–3) tended to be associated with a large number of people. However, the LST of compact high-rise/mid-rise/low-rise (i.e., LCZ 1–3) in summer was between 39.76 °C and 41.31 °C, which could be harmful to human health. Moreover, it was found again that the LCZ class with the maximum LST value was large low-rise (i.e., LCZ 8) for all seasons, which is consistent with the result of [43]. This was mainly because large low-rise (i.e., LCZ 8) areas contained clusters of factories that generated heat. Moreover, Budhiraja et al. also confirmed that LCZ 8 was the most heat-stressed LCZ class [42]. Conversely, the LCZ class with the minimum LST value in all seasons was observed to be water (i.e., LCZ G), which contrasts the results of [43]. A single image in a season was used to produce the LST map for this season in [43], which means that it may lack some representation when compared with the LST map derived from multi-temporal images in this paper. LCZ G, with the minimum LST value, illustrated that water had a good cooling effect. The LST values of dense trees (i.e., LCZ A) were lower than those of scattered trees (i.e., LCZ B) in each season, which indicated that there was a negative correlation between vegetation density and LST. The thicker the vegetation, the better the cooling effect. Bare surfaces, however, such as bare rock and road (i.e., LCZ E), had a relatively high LST value. This was mainly because most roads were located in the urban center, with high LST values, and the rock, soil, and sand were bare and not covered with trees or vegetation, leading to high heat accumulation. 

**Figure 5:**
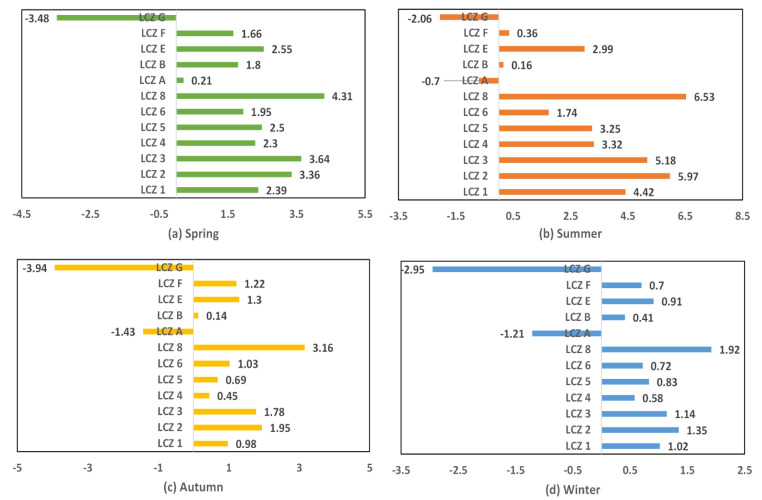
SUHI intensity in °C per LCZ class compared to LCZ D for all seasons.

The seasonal SUHI intensities were calculated by the difference per LCZ class and LCZ D (i.e., low plant), as shown in Figure 5. The maximum SUHI intensities of the LCZ 1–5, LCZ 8, and LCZ E classes occurred in summer. The maximum SUHI intensities of the LCZ A-B and LCZ F-G classes occurred in spring or autumn. This was mainly because the LCZ 1–5, LCZ 8, and LCZ E classes were urban or urban-related LCZ classes that had poor heat capacity. When the temperature rose in summer, these LCZ classes heated up quickly and easily. In contrast, the LCZ A-B and LCZ F-G classes were natural LCZ classes that had a relatively higher heat capacity than urban or urban-related LCZ classes. Therefore, these natural LCZ classes tended not to heat up easily. In terms of negative SUHI intensity, the LST difference between LCZ G (i.e., water) and LCZ D (i.e., low plant) was less than zero in all seasons, which indicated that the LST of water was always lower than that of low plant throughout the whole year; Except for the spring, the LST difference between LCZ A (i.e., dense tree) and LCZ D (i.e., low plant) was less than zero in all seasons. In terms of positive SUHI intensity, the LST differences between LCZ 1–6, LCZ 8, LCZ B, LCZ E-F, and LCZ D (i.e., low plants) were greater than zero. In addition, the range of SUHI intensity for the built-type LCZ classes (i.e., LCZ 1–6 and LCZ 8) was 1.95 °C to 4.31 °C in spring, 1.74 °C to 6.53 °C in summer, 0.45 °C to 3.16 °C in autumn, and 0.58 °C to 1.92 °C in winter. The maximum SUHI intensity was 6.53 °C in summer, which proves again that the SUHI effect was more significant in summer than in other seasons. Moreover, the maximum SUHI intensities were derived from the LST difference between LCZ 8 (i.e., large low-rise) and LCZ D (i.e., low plants). 

### 4.3. Seasonal SUHI Intensity Derived from the Urban–Rural Dichotomy

As seen in Table 4, the SUHI intensities using the urban–rural dichotomy in spring, summer, autumn, and winter were 1.32 °C, 3.15 °C, 1.05 °C, and 1.15 °C, respectively. The SUHI intensity was particularly strong in summer and the weakest in winter. This proves that again the SUHI effect was very significant in summer. Compared with the SHUI intensity derived from LCZ classification, the maximum SUHI intensity value derived from the urban–rural dichotomy was less than that derived from LCZ classification (i.e., 6.53 °C). The urban–rural dichotomy is a rough division method and may have some limitations in terms of reflecting a relatively strong SUHI intensity. The advantage of the traditional urban–rural dichotomy its simplicity of calculation. Similarly, the SUHI information obtained from this method is also simple. The SUHI intensity derived from the urban–rural dichotomy only showed the average LST values of urban and rural areas, which were divided based on the experience of researchers. Consequently, different researchers would produce different classifications of SUHI intensity. Hence, the SUHI intensity derived from the urban–rural dichotomy is empirical and unstable, so it can make a limited contribution to SUHI studies. In contrast, LCZ classification provides a standard method to conduct SUHI studies, which would produce more objective and stable SUHI intensity results. 

## 5. Conclusions

This paper analyzed the seasonal SUHI effect with LCZ classification and traditional urban–rural dichotomy methods in Wuhan based on the GEE platform. It can be concluded that (1) large low-rise (i.e., LCZ 8) had the maximum LST value in all seasons; (2) water (i.e., LCZ G) had the minimum LST value in all seasons; (3) the LST values of compact high-rise/midrise/low-rise (i.e., LCZ 1–3) were higher than those of open high-rise/midrise/low-rise (i.e., LCZ 4–6) in all seasons, and the LST values of dense trees (i.e., LCZ A) were lower than those of scattered trees (i.e., LCZ B) in all seasons; (4) summer had the strongest SUHI effect, followed by spring, autumn, and winter; (5) the maximum SUHI intensities derived from LCZ classification and the urban–rural dichotomy are 6.53 °C and 3.15 °C, respectively. 

This paper can provide some valuable information regarding the SUHI effect in Wuhan, contributing to urban sustainable development. The government should take measures to alleviate the SUHI effect in summer in future urban planning, such as increasing urban greening in compact high/mid/low-rise LCZ areas; improving watering frequency in summer; increasing the utilization rate of new energy vehicles and public transportation, and limiting heat emissions from factories, especially near the urban center. Overall, LCZ classification represents a more refined classification of the urban thermal environment and results in more accurate SUHI intensity estimation. With the support of the GEE platform, this paper provides a research framework that can be applied to other study areas to obtain more accurate and valuable information about the SUHI effect.

For further improvement in SUHI research with LCZ classification, it may be possible to further enhance the LCZ mapping accuracy. For example, other types of remote sensing imagery may provide useful information to refine the LCZ mapping, especially in relation to the built-type LCZ classes. In addition, the timing of LST maps derived from Landsat images is around 10:00 a.m. local time, which is not ideal for SUHI analysis. Hence, it would lower to some extent the SUHI intensity in this study. In further research, LST data closer to the ideal timing of SUHI intensity analysis are expected to be combined with a more accurate LCZ map to better understand SUHI intensity. 

## Figures and Tables

**Table 1:** The LCZ classification scheme [22].

LCZ Class		Definition	Sample Graph
Built type	LCZ 1: compact high-rise	Dense mix of tall buildings up to tens of stories. Few or no trees. Land cover mostly paved. Concrete, steel, stone, and glass construction materials.	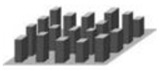
	LCZ 2: compact midrise	Dense mix of midrise buildings (3–9 stories). Few or no trees. Land cover mostly paved. Stone, brick, tile, and concrete construction materials.	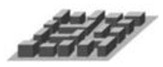
	LCZ 3: compact low-rise	Dense mix of low-rise buildings (1–3 stories). Few or no trees. Land cover mostly paved. Stone, brick, tile, and concrete construction materials.	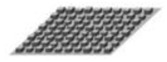
	LCZ 4: open high-rise	Open arrangement of tall buildings with tens of stories. Abundance of pervious land cover (low plants, scattered trees). Concrete, steel, stone, and glass construction materials.	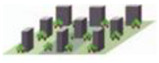
	LCZ 5: open midrise	Open arrangement of midrise buildings (3–9 stories). Abundance of pervious land cover (low plants, scattered trees). Concrete, steel, stone, and glass construction materials.	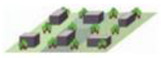
	LCZ 6: open low-rise	Open arrangement of low-rise buildings (1–3 stories). Abundance of pervious land cover (low plants, scattered trees). Wood, brick, stone, tile, and concrete construction materials.	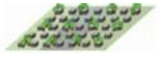
	LCZ 7: lightweight low-rise	Dense mix of single-story buildings. Few or no trees. Land cover mostly hard-packed. Lightweight construction materials (e.g., wood, thatch, corrugated metal).	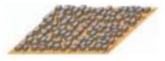
	LCZ 8: large low-rise	Open arrangement of large low-rise buildings (1–3 stories). Few or no trees. Land cover mostly paved. Steel, concrete, metal, and stone construction materials.	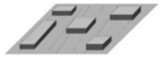
	LCZ 9: sparsely built	Sparse arrangement of small or medium-sized buildings in a natural setting. Abundance of pervious land cover (low plants, scattered trees).	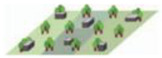
	LCZ 10: heavy industry	Low-rise and midrise industrial structures (towers, tanks, stacks). Few or no trees. Land cover mostly paved or hard-packed. Metal, steel, and concrete construction materials.	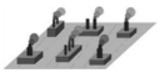
Land cover type	LCZ A: dense trees	Heavily wooded landscape of deciduous and/or evergreen trees. Land cover mostly pervious (low plants). Zone function is natural forest, tree cultivation, or urban park.	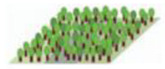
	LCZ B: scattered trees	Lightly wooded landscape of deciduous and/or evergreen trees. Land cover mostly pervious (low plants). Zone function is natural forest, tree cultivation, or urban park.	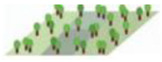
	LCZ C: Bush, scrub	Open arrangement of bushes, shrubs, and short, woody trees. Land cover mostly pervious (bare soil or sand). Zone function is natural scrubland or agriculture.	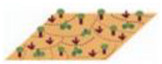
	LCZ D: low plants	Featureless landscape of grass or herbaceous plants/crops. Few or no trees. Zone function is natural grassland, agriculture, or urban park.	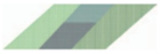
	LCZ E: bare rock or paved	Featureless landscape of rock or paved cover. Few or no trees or plants. Zone function is natural desert (rock) or urban transportation.	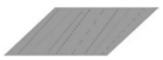
	LCZ F: bare soil or sand	Featureless landscape of soil or sand cover. Few or no trees or plants. Zone function is natural desert or agriculture.	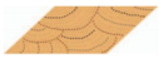
	LCZ G: water	Large, open water bodies such as seas and lakes, or small bodies such as rivers, reservoirs, and lagoons.	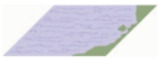

Source: Stewart and Oke (2012). ©American Meteorological Society. Used with permission. Note: the Local Climate Zone (LCZ) classes in red text were selected for LCZ classification in Wuhan.

**Table 2:** Confusion matrix of LCZ classes.

LCZ	1	2	3	4	5	6	8	A	B	D	E	F	G	Total	PA (%)
**1**	1215	118	44	1100	162	92	26	0	0	0	18	2	0	2777	43.75
**2**	142	1548	222	86	490	140	52	0	0	0	23	6	0	2709	57.14
**3**	4	62	621	5	65	28	0	0	0	0	4	1	0	790	78.61
**4**	986	22	10	1339	1144	34	26	2	0	0	31	3	0	3597	37.23
**5**	230	42	102	152	939	178	8	2	0	0	24	0	0	1677	55.99
**6**	102	13	32	32	19	1362	5	1	0	2	33	15	0	1616	84.28
**8**	79	19	11	16	29	4	5411	0	0	0	117	3	0	5689	95.11
**A**	0	0	1	1	15	13	0	3502	150	61	6	0	0	3749	93.41
**B**	3	0	0	0	1	50	0	310	1035	725	13	86	0	2223	46.56
**D**	13	0	0	2	0	1	0	0	3	1422	63	109	0	1613	88.16
**E**	47	48	37	30	10	59	161	0	0	0	4535	338	0	5265	86.13
**F**	0	0	1	0	0	17	0	0	10	123	0	2096	0	2247	93.28
**G**	0	0	0	0	0	0	0	0	0	0	1	0	4262	4263	99.98
**Total**	2821	1872	1081	2763	2874	1978	5689	3817	1198	2333	4868	2659	4262	38215	
**UA (%)**	43.07	82.69	57.45	48.46	32.67	68.86	95.11	91.75	86.39	60.95	93.16	78.83	100.00		
**OA (%)**	76.64	**OAb (%)**		65.18	**OAlc (%)**		88.06								

Note: OA = overall accuracy; PA = producers’ accuracy; UA = users’ accuracy; OAb = overall accuracy for built-type LCZ classes; OAlc = overall accuracy for land-cover-type LCZ classes.

**Table 3:** The average LST results of local climate zones in each season.

Class	Name	Average LST (°C)			
		Spring	Summer	Autumn	Winter
LCZ 1	Compact high-rise	24.16	39.76	25.64	13.82
LCZ 2	Compact midrise	25.13	41.31	26.61	14.15
LCZ 3	Compact low-rise	25.41	40.52	26.44	13.94
LCZ 4	Open high-rise	24.07	38.66	25.11	13.38
LCZ 5	Open midrise	24.27	38.59	25.35	13.63
LCZ 6	Open low-rise	23.72	37.08	25.69	13.52
LCZ 8	Large low-rise	26.08	41.87	27.82	14.72
LCZ A	Dense trees	21.98	34.64	23.23	11.59
LCZ B	Scattered trees	23.57	35.50	24.80	13.21
LCZ D	Low plant	21.77	35.34	24.66	12.80
LCZ E	Bare rock and paved	24.32	38.33	25.96	13.71
LCZ F	Bare soil and sand	23.43	35.70	25.88	13.50
LCZ G	Water	18.29	33.28	20.72	9.85

**Table 4:** The results of SUHI intensity using the urban–rural dichotomy.

Average Temperature (°C)	Spring	Summer	Autumn	Winter
Urban area	24.00	38.70	25.60	13.81
Rural area	22.68	35.55	24.56	12.65
SUHI intensity	1.32	3.15	1.05	1.15

## Data Availability

Data is available upon reasonable request from the corresponding author.
